# A new method to quantify the effect of co-medication on the efficacy of abiraterone in metastatic castration-resistant prostate cancer patients

**DOI:** 10.3389/fphar.2023.1220457

**Published:** 2023-09-28

**Authors:** Bertalan Fekete, Lili Bársony, Krisztina Biró, Fruzsina Gyergyay, Lajos Géczi, Attila Patócs, Barna Budai

**Affiliations:** ^1^ Central Hospital of Northern Pest, Budapest, Hungary; ^2^ Department of Laboratory Medicine, Semmelweis University, Budapest, Hungary; ^3^ Department of Genitourinary Medical Oncology and Clinical Pharmacology, Comprehensive Cancer Center, National Institute of Oncology, Budapest, Hungary; ^4^ Department of Molecular Genetics, Comprehensive Cancer Center, National Institute of Oncology, Budapest, Hungary; ^5^ National Tumor Biology Laboratory, Comprehensive Cancer Center, National Institute of Oncology, Budapest, Hungary

**Keywords:** abiraterone acetate, co-medication, drug-drug interactions, metastatic castration-resistant prostate cancer, overall survival, treatment duration

## Abstract

**Background and Objective:** Patients with metastatic castration-resistant prostate cancer (mCRPC) treated with abiraterone acetate (AA) have co-morbidities treated with different drugs. The aim was to quantify the potential effect of co-medications on AA treatment duration (TD) and overall survival (OS).

**Methods:** A new parameter, called “individual drug score” (IDS) was calculated by summing the “drug score”-s (DS) of all co-medications for each patient. The DS was determined by quantifying the effect of a given co-drug on enzymes involved in steroidogenesis and metabolism of AA. The correlation between log (IDS) and TD was tested by non-linear curve fit. Kaplan-Meier method and multivariate Cox regression was used for analysis of TD and OS.

**Results:** The IDS and TD of AA+prednisolone showed a dose-response correlation (*n* = 166). Patients with high IDS had significantly longer TD and OS (*p* <0.001). In multivariate analysis IDS proved to be an independent marker of TD and OS. The same analysis was performed in a separate group of 81 patients receiving AA+dexamethasone treatment. The previously observed relationships were observed again between IDS and TD or OS. After combining the AA+prednisolone and AA+dexamethasone groups, analysis of the IDS composition showed that patients in the high IDS group not only used more drugs (*p* <0.001), but their drugs also had a higher mean DS (*p* = 0.001).

**Conclusion:** The more co-drugs with high DS, the longer the duration of AA treatment and OS, emphasizing the need for careful co-medication planning in patients with mCRPC treated with AA. It is recommended that, where possible, co-medication should be modified to minimize the number of drugs with negative DS and increase the number of drugs with high DS. Our new model can presumably be adapted to other drugs and other cancer types (or other diseases).

## 1 Introduction

According to the WHO statistics in Hungary the incidence of prostate cancer (PC) is the first among all malignancies in men (6,234 new cases identified in 2020) and the age-adjusted mortality of prostate cancer is the third highest among malignant diseases in men (Sung et al., 2021). For almost all men who die of PC, mCRPC will be the cause of death. A variety of therapies have been approved for treatment of mCRPC by the European Medicines Agency, with the goal of improved efficacy outcomes. Abiraterone, cabazitaxel, enzalutamide, ^223^Ra dichloride, olaparib and ^177^Lu-PSMA-617 are administered to mCRPC patients (Biró et al., 2018; Tombal, 2023). The mean age of patients with mCRPC is 70 years, and more than 75% of these patients have at least one comorbid condition, but more than 30% have moderate or severe comorbidity (Zist et al., 2015). The comorbidities require additional pharmacological treatments which increase the chances of drug-drug interactions (DDI). Such interactions may occur in mCRPC patients due to the inhibitory effect of abiraterone acetate (AA) on liver cytochrome P450 (CYP)-dependent enzymes, which are also involved in the metabolism of other drugs (Del Re et al., 2017).

The AA is converted to abiraterone through hydrolysis. Thereafter, abiraterone is extensively metabolized primarily by sulfotransferase 2A1, and by CYP3A4 to inactive metabolites. The inhibited CYP enzimes are the followings: 1A2 (strong); 2C8 (weak); 2C9 (moderate); 2C19 (moderate); 2D6 (strong); 3A4 (moderate) (Benoist et al., 2016).

The level of steroidogenesis can determine the amount of ligand for androgen receptors (AR), and thus the inhibitory effect of AA on the synthesis of AR-ligand can be competitively increased or decreased by some drugs.

We hypothesized that besides AA, any co-administered drugs involved in the above described DDI and/or steroidogenesis could modify the efficacy of AA treatment.

There are some reports about DDIs involving AA. Statins can increase the efficacy of AA treatment (Harshman et al., 2017; Gordon et al., 2018; Wilson et al., 2022). Carbamazepine has an inverse effect, decreases the exposure to AA (Benoist et al., 2018). Indomethacin reduces prostate cancer cell growth and improves the response to AA (Liu et al., 2017). The use of angiotensin system inhibitors (ASI) may enhance and prolong AA therapy (Wilk et al., 2021). Rifampicin decreased AA exposure by half (Bernard et al., 2015).

There are several types of drug-drug interactions. The main pharmacokinetic interactions involve drug metabolism by the CYP enzyme system. This system has several enzymes, including CYP3A4, which is most often involved in drug metabolism. Other CYP enzymes (2D6, 1A2, 2C8, 2C9, 2C19, etc.) can also participate in this system. The pharmacokinetic interaction between two drugs may involve different mechanisms: 1) Drugs A and B are metabolized by the same CYP enzyme and thus compete, resulting in a reduction or slowdown of the metabolism of A, B, or both. 2) Drug A inhibits the metabolism of drug B, leading to the risk of an overdose by drug B due to the reduction of its metabolism. 3) Drug A induces the metabolism of drug B causing subtherapeutic concentrations of drug B by its accelerated metabolism. It is also important to note that the cytochrome P450 enzymes are present in most body tissues (Tsoukalas et al., 2022).

While AA inhibits androgen synthesis by blocking CYP17A1 activity, androgen production is not completely abrogated. Intratumoral androgens are hypothesized to originate from multiple sources. *De novo* dihydrotestosterone (DHT), the main androgen, is synthesized from cholesterol through to testosterone, which is the classical biosynthesis pathway, but it can also be synthesized through a “backdoor pathway” that does not require testosterone as a precursor. Here androsterone serves as the main substrate. As both of these pathways utilize cholesterol as a starting substrate, increased levels of cholesterol could promote androgen synthesis. Another potential source contributing to intracrine androgen synthesis is the presence of DHEA sulfate (DHEA-S). DHEA-S is present at plasma concentrations up to 500 times higher than testosterone. Conversion of circulating DHEA-S into DHEA is an alternate source of androgen. In prostate cancer patients treated with AA, a significant circulating DHEA-S concentration is still present (Armstrong and Gao, 2021).

We hypothesized that besides AA, any co-administered drugs involved in the above described drug-drug interaction and/or androgenesis could modify the efficacy of AA treatment. The aim of this study was to test whether consequent co-medication of patients receiving AA treatment has any effect on disease progression, which is reflected by the duration of AA treatment. The overall survival was also investigated. During AA treatment, patients also receive a corticosteroid, typically prednisone/prednisolone or dexamethasone (Yang et al., 2021). Therefore, two separate groups treated with AA were studied: those who received only prednisolone, and those who received dexamethasone.

As the literature reported that neutrophil-to-lymphocyte ratio (NLR) (Guan et al., 2020; Nieblas-Toscano et al., 2020), platelet-to-lymphocyte ratio (PLR) (Lolli et al., 2016; Guan et al., 2020), systemic immune-inflammation index (SII) (Lolli et al., 2016), prognostic nutritional index (PNI) (Fan et al., 2017; Küçükarda et al., 2022) and prostate-specific antigen (PSA) dynamics (Cindolo et al., 2017; Iacovelli et al., 2022) can be predictive factors of AA treatment, we also aimed to study the effect of these derived parameters on TD and OS.

## 2 Patients and methods

### 2.1 Patients

All consecutive patients with mCRPC treated between 2011 May and 2022 January were enrolled and their data were retrospectively analyzed. Patients were sorted into two groups depending on corticosteroid (prednisolone or dexamethasone) supplementation. The exploration group included patients treated with AA+prednisolone, while in the validation group the patients were treated with AA+dexamethasone, or during the AA therapy a switch from prednisolone to dexamethasone was applied. Optimally, this second group should only include the AA+dexamethasone receiving patients, but such cases were few, therefore, we included those who switched from prednisolone to dexamethasone at the first PSA elevation. These groups are further referred to as AA+prednisolone and AA+dexamethasone groups, respectively.

Inclusion criteria: presence of mCRPC; pre-chemotherapy or post-chemotherapy AA treatment. Exclusion criteria: treatment cessation because of non AA-related adverse events or patients’ request; brain metastases; other parallel malignancies; insufficient data regarding co-medications or follow-up; ongoing AA treatment.

In case of co-morbidities, the criteria for starting AA treatment included: adequate liver, kidney and heart functions.

The Institutional Ethical Committee and the Hungarian Medical Research Council approved the study (323-101/2005-1018EKU). All patients signed an informed consent.

### 2.2 Treatment

AA+prednisolone was administered according to the treatment protocol including 1,000 mg AA and 10 mg prednisolone daily. The prednisolone can be changed to 0.5 mg dexamethasone when serum level of PSA increased and no other progression was present (Yang et al., 2021). A few patients to whom dexamethasone was administered before the AA treatment (Venkitaraman et al., 2008) started with the AA+dexamethasone combination (Attard et al., 2019). The AA treatment was covered until 2 of the 3 progressions (PSA, imaging, clinical) appeared.

The names of all drugs taken by patients during the entire AA treatment duration (including oncologic and other co-medications) were extracted from the electronic database of the health informatics system.

The locations of metastases were recorded before the AA treatment.

Follow-up of all patients was performed every 3 months and included physical examination, abdominal ultrasonography, CT, MRI or PET-CT, and laboratory evaluation of hematological parameters, liver and kidney functions and PSA determination. The systemic treatments after AA were also recorded.

### 2.3 Calculation of drug score (DS) and individual drug score (IDS)

A database of taken drugs was constructed in Microsoft Excel. All drugs have been screened for interactions with CYP1A2, CYP2C8, CYP2C9, CYP2C19, CYP2D6 and CYP3A4 enzymes, which may interact with AA according to the data of Drug Categories section of Drugbank (Wishart et al., 2018) (https://go.drugbank.com/drugs/DB05812). For a given drug, taking into account the data provided in the Drug Categories section of Drugbank Interaction Checker (https://go.drugbank.com/drug-interaction-checker) for that drug, a positive score is given for each enzyme if the drug is metabolized or inhibited by the enzymes listed. If a drug induces any enzyme, then will receive a negative score for each involved enzyme. If the strength of the interaction is weak, moderate (or unknown) or strong, the score is considered to be 1, 2 or 4. This score was summed for each drug.

The influence of drugs on steroidogenesis was extracted from the literature (see Supplementary Table S1) including human trials using the drug name and the following keywords: testosterone, dehydroepiandrosterone and cholesterol. An inhibitory effect was scored as 1 point, increased steroidogenesis was scored as −1 point, and 0 points were scored for no effect or no data. This score was added to the sum calculated above.

**FIGURE 1:**
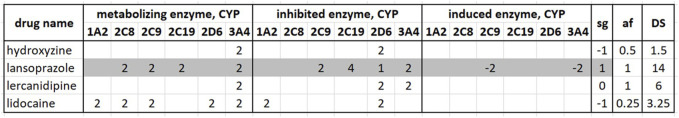
Drug score (DS) for medicines used during the abiraterone acetate (AA) treatment (a representative part of Supplementary Table S1). DS calculation method: the scores from the grey cells are summed, then multiplied by the drug administration factor (af) (see more details in the Methods section). CYP, cytochrome P450; sg, steroidogenesis.

The duration of drug administration was also considered and a multiplier (af) was applied to the final score. If a drug was used continuously, intermittently, or rarely, the af of 1, 0.5, and 0.25 were used. The final DS of each drug is presented in the Supplementary Table S1. A representative part of the Supplementary Table S1 and the DS calculation is presented in Figure 1. For each patient the final IDS, which was used for further analysis, was calculated by summing the DS of each taken drug.

For each patient a sum of DS was calculated based on every taken medicine resulting in the final IDS value, which was used for further analyses.

### 2.4 Laboratory measurements

Blood tests were taken at the start of AA therapy. PSA was also tested 1 month and 3 months after the start of AA. Plasma levels of lymphocytes, neutrophils, platelet count and albumin concentration were registered to calculate the NLR, PLR, SII (platelet count*NLR), PNI (albumin*lymphocyte). Serum PSA level was determined using LIAISON^®^ PSA ILMA kit (DiaSorin S.p.A., Saluggia, Italy).

### 2.5 Statistics

The primary objective was the TD of AA. The secondary objective was the OS calculated from the start of AA until prostate cancer-related death or the last follow-up. A further objective was to assess the predictive role of some derived laboratory parameters.

The IDS was considered as a pseudo AA concentration, thus the log (IDS) was tested for fit with a non-linear dose-response curve with variable slope (four parameters) using the GraphPad Prism 6 software. The response in this case was the TD. The threshold for dichotomization of IDS was the lowest effective IDS calculated according to (EFSA et al., 2017) considering 10% benchmark response for non-continuous data.

The median was the cut-off for dichotomization of parameters, except Gleason score. The PSA change was defined as a ≥50% decrease at 3 months in the PSA level relative to the starting level (Cindolo et al., 2017). For univariate analysis of TD and OS Kaplan-Meier method and the log-rank test was used. Significant (*p* <0.05) variables in the univariate analysis were included in multivariate Cox regression analyses; in order to avoid multicollinearity, separate analyses were performed and if necessary some variables were excluded. The NCSS 2019 Statistical Software (NCSS, LLC. Kaysville, Utah, United States, ncss.com/software/ncss) was used for statistical analyses.

## 3 Results

In total 351 patients were enrolled, but after critical review of available data 104 were excluded for the following reasons: 34 ongoing AA treatment, 32 treatment cessations because of non AA-related adverse events or patients’ request, 18 insufficient data regarding co-medications or follow-up, 11 brain metastases, 9 with other parallel malignancies. The remaining 247 patients were separated in the AA+prednisolone (*n* = 166) and AA+dexamethasone (*n* = 81) groups.

**FIGURE 2:**
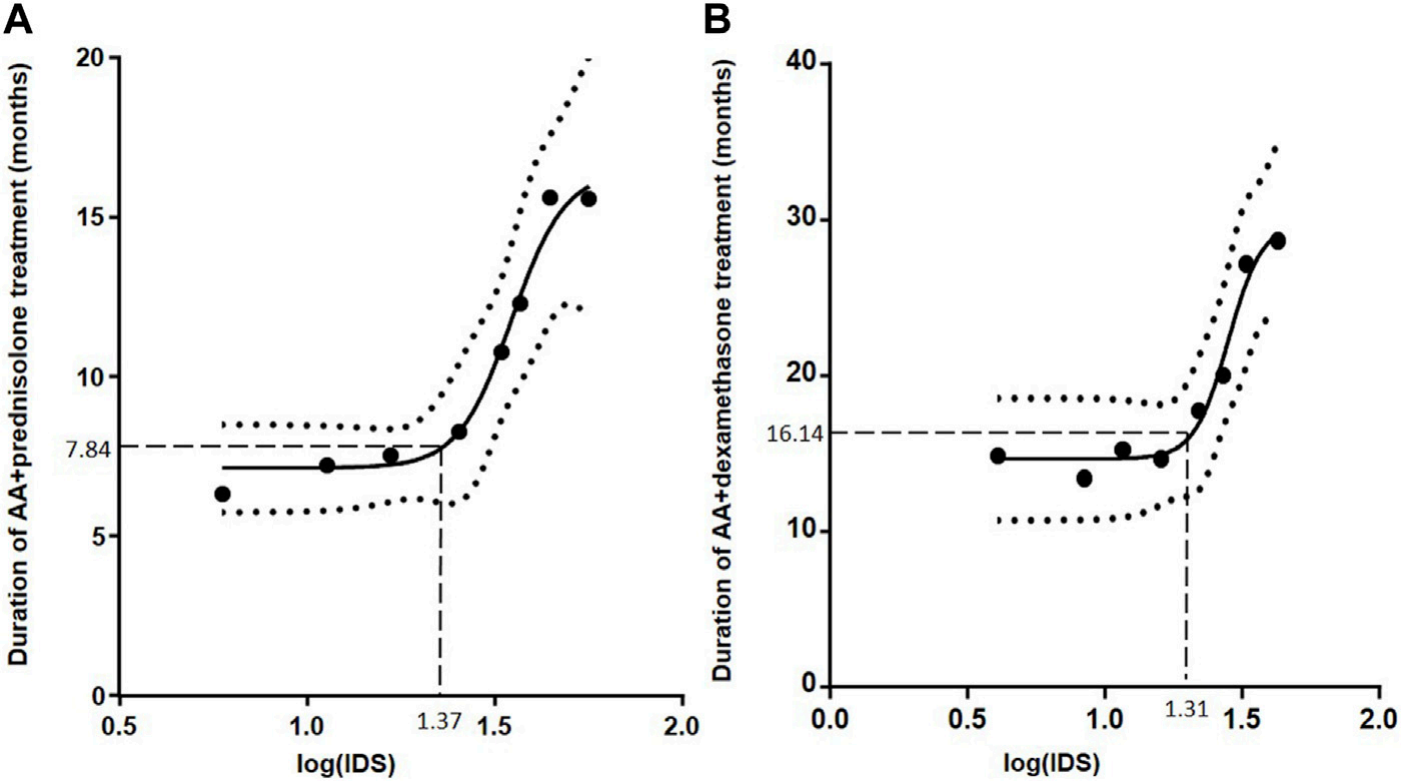
Influence of individual drug score (IDS) on treatment duration (TD) of abiraterone acetate (AA)+prednisolone **(A)** and AA+dexamethasone **(B)**. The curve (black line) was calculated with a non-linear dose-response fit. The dotted curves represent the 95% confidence range for the fitted curve. The cut-off level of IDS was determined (dashed lines) at no observed effect level (bottom plateau) + 10%, resulting in an IDS cut-off level of 23.4 for AA+prednisolone **(A)** and 20.5 for AA+dexamethasone **(B)**

The correlation between the IDS and TD of AA+prednisolone is presented in Figure 2A.

The characteristics of patients on AA+prednisolone are presented in Table 1.

**Table 1:** Clinico-pathologic characteristics of patients on abiraterone acetate+prednisolone treatment and univariate analysis of treatment duration (TD) and of overall survival (OS).

Parameters	N (%)	mTD (95% CI) months	*p*	mOS (95% CI) months	*p*
	166	6.5 (5.6-8.1)		16.9 (13.4-18.8)	
Age median (range) years	72 (49-90)				
<72	78 (47)	7.7 (6.3-8.7)	0.31	17.6 (14.4-20.7)	0.45
≥72	88 (53)	5.6 (5.3-7.3)		14.4 (13-19.4)	
Gleason score (NA n=26)					
≤7	54 (33)	7.0 (5.5-8.3)	0.93	18.5 (14.2-20.7)	0.87
>7	86 (52)	5.9 (5.5-8.5)		17.3 (14-22)	
Previous chemotherapy					
yes	131 (79)	6.9 (5.7-8.1)	0.97	16.9 (13.3-18.7)	0.38
No	35 (21)	6.5 (3.9-9.8)		16.4 (9.7-23.7)	
Site of metastases before treatment					
bone only	46 (28)	8.3 (5.5-9.8)	0.24	21.4 (18.3-25)	0.18
bone+lymph node	50 (30)	7.3 (5.9-8.7)		14.4 (13-20.1)	
bone+lymph node+visceral	33 (20)	5.8 (4.4-7.2)		12.3 (8-14.4)	
bone+other	23 (14)	5.5 (3.7-8.0)		15.6 (10.5-18.5)	
lymph node only	7 (4)	5.7 (2.5-8.6)		14.4 (11.7-20.8)	
lymph node+visceral	6 (4)	7.0 (5.9-7.2)		17.1 (16.9-29.8)	
visceral	1 (1)	3.1		22.6	
Multiple metastases					
yes	74 (45)	5.9 (5.4-8.9)	0.36	20.5 (17-22.4)	0.13
no	92 (55)	6.7 (5.6-7.7)		14 (12.3-16.4)	
PSA mean (95% CI) ng/ml					
before treatment (n=166)	313 (208-417)				
after 1 month (n=134)	288 (112-413)				
after 3 months (n=144)	212 (137-288)				
PSA change (NA n=22)					
<50% after 3 months	91 (55)	6.2 (5.4-7.2)	<0.001	17 (13.4-19)	0.01
≥50% after 3 months	53 (32)	10.0 (8.5-12.4)		22.6 (17.1-25.6)	
LDH median (95% CI) U/l (NA n=23)	446 (424-482)				
<446	71 (43)	8.5 (5.7-9.2)	0.01	22.6 (18-25.5)	<0.001
≥446	72 (43)	5.6 (4.6-7.40)		12.7 (10.2-15.8)	
NLR median (95% CI) (NA n=28)	3.1 (2.7-3.5)				
<3.1	71 (43)	8.1 (5.7-9.1)	0.003	19.4 (14.4-22.9)	<0.001
≥3.1	70 (42)	5.4 (3.9-5.8)		12.7 (10.5-16.9)	
SII median (95% CI) (NA n=28)	734 (661-850)				
<734	69 (42)	5.9 (5.2-7.2)	0.85	15.8 (13-19)	0.13
≥734	69 (42)	5.9 (5.4-8.7)		16.4 (12.5-18.7)	
PLR median (95% CI) (NA n=28)	165 (150-178)				
<165	69 (42)	7.2 (5.4-8.9)	0.10	17.3 (13-20.1)	0.02
≥165	69 (42)	5.6 (5.2-6.9)		14.6 (10.5-17.6)	
PNI median (95% CI) (NA n=62)	65 (59-69)				
<67	52 (31)	5.8 (5.2-7.7)	0.11	13 (10.2-18)	0.01
≥67	52 (31)	8.6 (5.7-9.4)		19.4 (14.7-24.2)	
IDS median (95% CI)	18.6 (15.5-20.7)				
<23.4	109 (66)	5.5 (5.1-6.4)	<0.001	13 (10.2-14.6)	<0.001
≥23.4	57 (34)	9.3 (7.7-10.3)		22.6 (19.4-27)	
Further line(s) of systemic therapy					
no	70 (42)	-		7.6 (5.9-9.2)	<0.001
yes	96 (58)			22.6 (18.8-25.6)	

CI, confidence interval; IDS, individual drug score; LDH, lactate dehydrogenase; mTD, median treatment duration; mOS, median overall survival; NA, not available; NLR, neutrophil-to-lymphocyte ratio; PNI, prognostic nutritional index; PRL, platelet-to-lymphocyte ratio; PSA, prostate-specific antigen; SII, systemic immune-inflammation index.

In univariate analysis, the median TD of AA+prednisolone was significantly influenced by 4 parameters, which were included in the multivariate Cox model. Besides the “PSA change” and NLR, the IDS proved to be an independent marker of TD (Table 2).

**TABLE 2:** Multivariate analysis of treatment duration (TD) and overall survival (OS).

Parameters	HR_TD_ (95% CI)	*p*	HR_OS_ _Cox1_ (95% CI)	*p*	HR_OS_ _Cox2_ (95% CI)	*p*
ABIRATERONE ACETATE + PREDNISOLONE						
PSA change						
<50% after 3 months	1 (ref.)	0.001	1 (ref.)	0.02	1 (ref.)	0.04
≥50% after 3 months	0.5 (0.4–0.8)		0.6 (0.4–0.9)		0.6 (0.4–1)	
LDH (U/l)						
<446	1 (ref.)	0.35	-		1 (ref.)	0.002
≥446	1.2 (0.8–1.8)				1.9 (1.3–3.9)	
NLR						
<3.1	1 (ref.)	0.002	1 (ref.)	<0.001	1 (ref.)	<0.001
≥3.1	1.8 (1.3–2.7)		2.1 (1.4–3.1)		2.2 (1.5–3.4)	
IDS						
<23.4	1 (ref.)	<0.001	-		1 (ref.)	0.01
≥23.4	0.4 (0.3–0.7)				0.6 (0.4–0.9)	
Further lines						
no	-		1 (ref.)	<0.001	-	
yes			0.3 (0.2–0.4)			
ABIRATERONE ACETATE + DEXAMETHASONE						
Gleason score						
≤7	1 (ref.)	0.11	1 (ref.)	0.02	1 (ref.)	0.20
≥7	1.5 (0.9–2.5)		2.3 (1.2–4.4)		1.6 (0.8–3.2)	
PSA change						
<50% after 3 months	1 (ref.)	0.01	1 (ref.)	<0.001	-	
≥50% after 3 months	0.5 (0.3–0.8)		0.2 (0.1–0.5)			
IDS						
<20.5	1 (ref.)	<0.001	-		1 (ref.)	0.002
≥20.5	0.4 (0.2–0.7)				0.3 (0.1–0.6)	
Age						
<71	-		1 (ref.)	0.001	1 (ref.)	0.01
≥71			3.4 (1.6–7.2)		2.4 (1.2–4.6)	
PLR						
<148	-		1 (ref.)	0.002	1 (ref.)	<0.001
≥148			3 (1.5–6.1)		4.1 (1.9–9)	
Further line(s)						
no	-		1 (ref.)	<0.001	1 (ref.)	<0.001
yes			0.2 (0.1–0.5)		0.2 (0.1–0.4)	

CI, confidence interval; HR, hazard ratio; IDS, individual drug score; LDH, lactate dehydrogenase; NLR, neutrophil-to-lymphocyte ratio; PLR, platelet-to-lymphocyte ratio; PSA, prostate-specific antigen; ref., reference.

In the case of OS, there were more significant parameters in the univariate analysis (Table 1). In order to avoid multicollinearity, IDS and “further lines” were tested separately (Cox1, Cox2) and PLR and PNI were excluded. Besides “PSA change”, LDH, NLR and “further lines”, the IDS was an independent marker of OS (Table 2).

In order to check our model, we analyzed the AA+dexamethasone group who received dexamethasone (*n* = 20) or changed prednisolone to dexamethasone (*n* = 61). The TD and OS did not differ between these 2 subgroups (data not shown). The correlation between the IDS and TD of AA+dexamethasone is presented in Figure 2B.

The characteristics of patients on AA+dexamethasone are presented in Table 3.

**TABLE 3:** Clinico-pathologic characteristics of patients on abiraterone acetate+dexamethasone treatment and univariate analysis of treatment duration (TD) and overall survival (OS).

Parameters	N (%)	mTD (95% CI) months	*p*	mOS (95% CI) months	*p*
	81	17.6 (14.9–20.3)		36.6 (32.2–42.4)	
Age median (range) years	71 (69–74)				
<71	40 (49)	21.7 (15.7–25.8)	0.07	43.8 (35.6–46.3)	0.01
≥71	41 (51)	16.9 (11.7–18.6)		29.4 (24.2–34.3)	
Gleason score (NA n = 9)					
≤7	29 (36)	20.3 (18.6–26.3)	0.03	41.7 (36.6–48)	0.02
>7	43 (53)	15.1 (12.4–17.5)		32.5 (28.6–35.6)	
Previous chemotherapy					
yes	54 (67)	18.6 (15–21.7)	0.30	36.6 (31.1–44.6)	0.54
no	27 (33)	17.5 (13.5–22)		34.3 (28.6–37.7)	
Site of metastases before treatment					
bone only	33 (41)	17.6 (14.3–21.7)	0.43	37.7 (32.5–44.6)	0.68
bone+lymph node	20 (25)	19.8 (11.8–26.9)		40.9 (28.6–48)	
bone+lymph node+visceral	10 (12)	9.2 (5.3–17.4)		19.1 (13.9–46.4)	
bone+visceral	9 (11)	15.7 (14–19.4)		35.6 (29.4–47.5)	
lymph node only	3 (4)	22.1 (17.2–24.5)		32.8 (32.8–32.9)	
lymph node+visceral	6 (7)	7.6 (6.1–12.4)		26 (10.2–52.8)	
Multiple metastases					
yes	45 (55)	7.5 (14.3–21.7)	0.36	32.9 (32.2–41.4)	0.34
no	36 (45)	18.6 (11.8–23.9)		41.7 (28.6–46.4)	
PSA mean (95% CI) ng/mL					
before treatment (n = 81)	120 (71–169)				
after 1 month (n = 78)	81 (31–131)				
after 3 months (n = 78)	68 (33–103)				
PSA change (NA n = 3)					
<50% after 3 months	29 (36)	11.7 (9.2–14.9)	<0.001	29.4 (18.3–32.5)	<0.001
≥50% after 3 months	49 (60)	22.1 (18.6–25.8)		43.8 (35.6–47.2)	
LDH median (95% CI) U/l (NA n = 13)	386 (355–420)				
<386	34 (42)	19.4 (14.8–23.9)	0.43	37.7 (29.4–42.4)	0.63
≥386	34 (42)	17.5 (11.8–22.1)		32.8 (26–44.6)	
NLR median (95% CI) (NA n = 10)	2.81 (2.7–3.5)				
<2.81	35 (43)	21.7 (14.8–25.6)	0.42	37.7 (32.2–44.8)	0.23
≥2.81	36 (44)	17.5 (9.6–19.4)		32.9 (24.2–41.4)	
SII median (95% CI) (NA n = 10)	621 (503–827)				
<621	35 (43)	22 (15–25.6)	0.21	40.9 (32.2–44.8)	0.23
≥621	36 (44)	17.2 (11.7–19.1)		32.8 (24.2–41.4)	
PLR median (95% CI) (NA n = 10)	148 (127–175)				
<148	35 (43)	23.6 (14–25.8)	0.13	37.7 (32.2–48)	0.01
≥148	36 (44)	17.5 (12.4–19.4)		32.8 (21.3–41.7)	
PNI median (95% CI) (NA = 10)	70 (55–88)				
<70	15 (19)	19.4 (17.6–21.7)	0.60	41.4 (21.3–47.5)	0.26
≥70	14 (17)	22.1 (12.2–25.8)		32.8 (31.1–36.6)	
IDS median (95% CI)	22.5 (18.5–25)				
<20.5	34 (42)	12.4 (9.6–14.9)	<0.001	31.1 (27.2–32.5)	0.049
≥20.5	47 (58)	19.8 (17.6–25.8)		41.7 (32.9–46.4)	
Further line(s) of systemic therapy					
no	28 (35)	-		26 (17.4–32.5)	<0.001
yes	53 (65)			43.8 (32.9–46,4)	

CI, confidence interval; IDS, individual drug score; LDH, lactate dehydrogenase; mTD, median treatment duration; mOS, median overall survival; NA, not available; NLR, neutrophil-to-lymphocyte ratio; PNI, prognostic nutritional index; PRL, platelet-to-lymphocyte ratio; PSA, prostate-specific antigen; SII, systemic immune-inflammation index.

In univariate analysis, the TD of AA+dexamethasone was significantly influenced by 3 parameters, which were included in the multivariate Cox model. “PSA change” and IDS proved to be independent markers of TD (Table 3). There were more significant parameters in the univariate analysis of OS (Table 3). In order to avoid multicollinearity “PSA change” and IDS were tested in separate Cox regression models (Cox1, Cox2). Besides “PSA change”, “Gleason score”, age, PLR and “further lines”, the IDS was an independent marker of OS (Table 2).

Beyond the threshold, the higher the IDS score is, the longer the TD lasts. The terciles of median TD in the low and high IDS groups were 6.3, 6.7, 6.4 months (*p* = 0.547) and 9.3, 15.7, 18.6 months (*p* = 0.043), respectively. This effect can also be observed in Figure 2.

**FIGURE 3:**
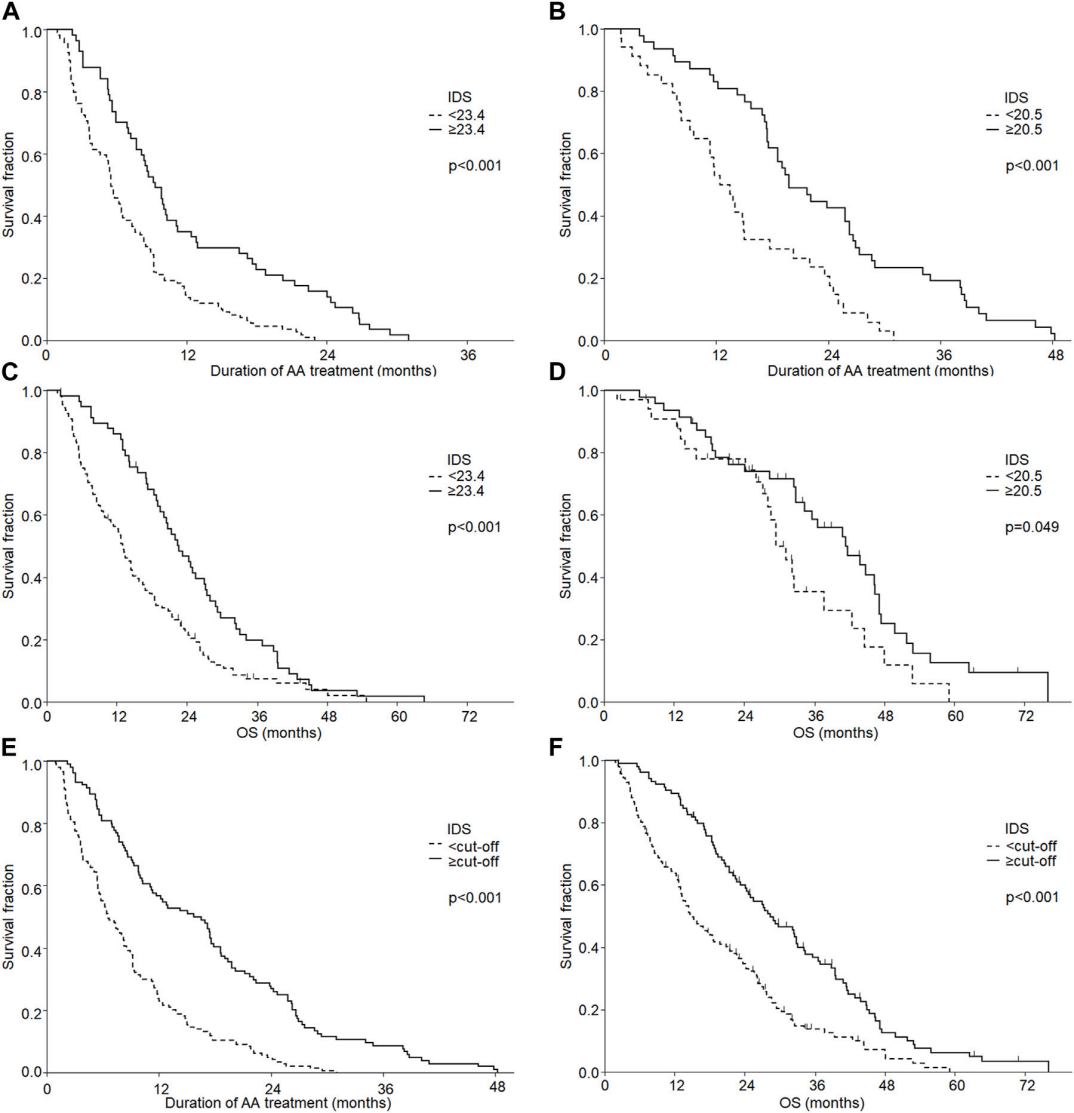
Effect of individual drug score (IDS) on treatment duration of abiraterone acetate (AA) **(A,B,E)** and overall survival (OS) **(C,D,F)** according to the used corticosteroid: prednisolone **(A,C)**, dexamethasone **(B,D)** and the combined group **(E,F)**

The high IDS proved to be an independent marker of longer TD and OS regardless of the corticosteroid used, therefore, the two groups were combined (Figure 3).

**FIGURE 4:**
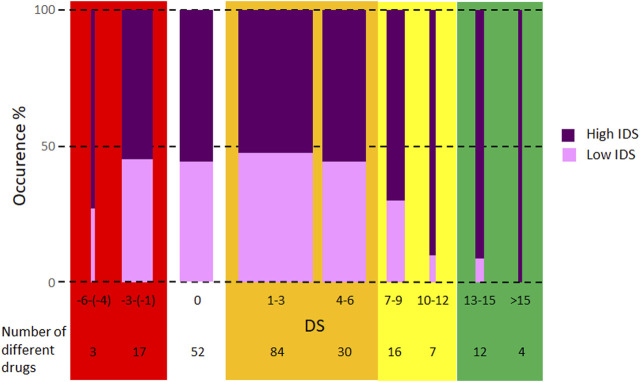
Prevalence of drugs in high and low individual drug score (IDS) groups. The width of the columns is proportional to the total prevalence of drugs in the group. Drug score (DS) values were rounded according to rounding rules, except ± 0.25, which was rounded to ± 1. The background colors were also used in Table 4 to aid co-medication planning.

Hereinafter, we analyzed the composition of IDS. Patients used 225 different drugs (Supplementary Table S1). Figure 4 shows the prevalence of drugs in the high and low IDS groups according to their DS values. Patients in the high IDS group used more drugs (prevalence >50%, *p* <0.001) in all DS categories presented. Drugs with high DS (>12) were used mainly by patients in the high IDS group (prevalence >90%, *p* <0.001). The prevalence of drugs with negative DS was not different in the high and low IDS groups (*p* = 0.9). Patients in the high IDS group not only used more drugs, but their drugs also had a higher mean DS (mean ± SD = 18.8 ± 48 vs. 12.7 ± 41 in the low IDS group, *p* = 0.001).

Based on these data it is suggested that the co-medication could be modified, if possible, to minimize the number of drugs with negative DS and to increase the number of drugs with high DS. Based on these considerations Table 4 has been prepared to assist the co-medication planning. In this table, comments from experts in different specialties for some group of drugs have been included. There are some groups of medicines where suggestions such as those given in Table 4 cannot be made because the co-morbidity requires the use of specific medicines, such as antibiotics, antidepressants and mood stabilizers. Even for these, the high DS medication should be preferred if there is an option. For groups of medicines not included in Table 4, some suggestions can be made: the choice of angiotensin II receptor blockers with high DS can be an alternative instead of angiotensin-converting-enzyme inhibitors (DS = 0–1); instead of rilmenidine (0) and moxonidine (0), calcium channel blockers with high DS or doxazosin (9) can be an option; zolpidem (10) is a suggested sleeping pill; for antihistamines, loratidine has a high DS (8.5); among non-opioid pain killers, diclofenac (5.5) is the best, most frequently chosen drug; budesonide (−6) should be avoided; vitamin D (5) was used many times, whereas the majority of other dietary supplements like vitamins, minerals and other bioactive components had little impact on IDS.

**TABLE 4:** Drug score (DS) for different drug types and remarks for co-medication planning. Involvement of co-specialists in planning should be considered.

ARBs	telmisartan	candesartan	valsartan	irbesartan	lossartan	Choose a drug with high DS (e.g., losartan, irbesartan) instead of telmisartan, while controlling blood pressure.								
DS	−1	4	5	9	13									
CaBs	lacidipine	amlodipine	lercanidipine	felodipine	nifedipine	diltiazem	verapamil	Instead of low-DS agents (e.g., lacidipine, amlodipine), choose a high-DS agent (e.g., felodipine, nifedipine, diltiazem) while controlling blood pressure.						
DS	2	4	6	11	12	17	17							
BBs	sotalol	bisoprolol	atenolol	betaxolol	timolol	metoprolol	nevibolol	carvedilol	Instead of low-DS agents (e.g., bisoprolol, atenolol), choose a high-DS agent (e.g., nebivolol, carvedilol) while controlling blood pressure and heart rate.					
DS	1	1	3	4	5	7	8	8						
ABs	prazosin	doxazosin	Choose doxazosin instead of prazosin, while controlling blood pressure.											
DS	0	9												
Diuretics	furosemide	spironolactone	clopamide	amiloride	chlortalidone	HCT	indapamide	etacrynic acid	eplerenone	triamterene	Instead of spironolactone, eplerenone is an option.			
DS	−1	−1	−1	0	0	1	1	1	2	3				
Antiarrhytmics	magnesium	digoxin	sotalol	digitoxin	lidocaine	propafenone	diltiazem	verapamil	amiodarone					
DS	−1	1	1	3	3.25	13	17	17	23					
Statins +	rosuvastatin	atorvastatin	simvastatin	fluvastatin		ezetimibe	For patients taking rosuvastatin, consideration should be given to switch to a statin with higher DS (e.g., atorvastatin). Of course, for those in whom combination cholesterol-lowering therapy (e.g., ezetimibe) is professionally justified, this will further increase the IDS.							
DS	5	12	13	15		5								
Fibrates	ciprofibrate	fenofibrate	Fenofibrate is an option to consider.											
DS	1	7												
Antiplatelets	aspirin	clopidogrel	Clopidogrel is the drug of choice.											
DS	1	14												
Anticoagulants	edoxaban	dabigatran	LMWH	aspirin	riveroxaban	acenocumarol	apixaban	warfarin	On the management of anticoagulation in patients with mCRPC receiving abiraterone, see Dubinsky et al., 2019.					
DS	0	0	1	1	2	8	10	13						
PPIs	esomeprazole	pantoprazole	omeprazole	rabeprazole	lansaprazole	Choose a high-DS drug (e.g., lansoprasol) instead of a low-DS drug (e.g., esomeprazole).								
DS	4	5	9	13	14									
H2RBs	famotidine	ranitidine	The choice of ranitidine instead of famotidine should be considered.											
DS	1	7												
Antidiabetics	insulin	vildagliptin	liraglutide	metformin	acarbose	gliclazide	gliquidone	saxagliptin	glimepiride	sitagliptin	linagliptin	dapaglifozin	glibenclamide	Here dapaglifozin is highlighted. Moreover, when it becomes necessary after metformin, instead of insulin, dapagliflozin may be recommended.
DS	−3	0	1	1	1	3	3	3	3	4	5	10	10	
Opioids	ethylmorphine	opiates	fentanyl	codeine	tramadol	oxycodone	Oxycodone is the drug of choice.							
DS	0.5	1.5	2	2.5	4	5								
Antiemetics	hyoscine	granisetron	metoclopramid	domperidon	ondansetron	cannabidiol	Ondansetron is the drug of choice.							
DS	0	1	3.5	4	10	10.5								

ABs, alpha blockers; ARBs, angiotensin II receptor blockers; BBs, beta blockers; CaBs, calcium channel blockers; H2RBs, histamine-2 receptor blockers; HCT, hydrochlorothiazide; IDS, individual drug score; LMWH, low molecular weight heparin; PPIs, proton pump inhibitors. The background color of DS, is similar to that used in Figure 4.

## 4 Discussion

In this study the additive effect of multiple co-medications on AA treatment efficacy was studied. Each taken drug, which modifies the androgenesis or interacts with CYP enzymes involved in the metabolism of AA, was scored (DS) and by summing the DS of all taken drugs during AA treatment, a final IDS for each patient was determined. The IDS varied in a wide range and after dichotomization the high IDS proved to predict a significantly longer AA treatment and OS compared to the low IDS. In case of patients treated with AA, the IDS is a mirror of DDI, which was the main subject of some reports (Graff and Beer, 2014; Benoist et al., 2016; Jamani et al., 2016; Bonnet et al., 2017; Del Re et al., 2017; Dubinsky et al., 2019; Escudero-Vilaplana et al., 2020; Vicente-Valor et al., 2021). However, none of these studies analyzed the impact of DDI on the outcome of AA treatment.

Four publications have retrospectively investigated the DDI in 72 (Escudero-Vilaplana et al., 2020), 95 (Bonnet et al., 2016), 84 (Jamani et al., 2016) and 87 (Vicente-Valor et al., 2021) mCRPC patients on AA treatment. They used search software of DDI databases (e.g., Lexicomp, Micromedex, etc.) to find potential DDI. According to a study of interactions between oral antineoplastic agents and concomitant medications (Escudero-Vilaplana et al., 2020) tamsulosin (18%) was the most frequent drug with potential DDI for prostate cancer. In our study tamsulosin (DS = 4) was the 19th in the frequency list and was used by 12% of all patients. In a study about DDI of AA (Bonnet et al., 2017), the most frequently used drug types were opioid pain killers (44%), beta-blockers (17%), and antiarrhythmics (9%). The most frequently used drugs having potential DDI with AA were nebivolol and tramadol (14%–14%). Opioid pain killers occurred in 43%, beta-blockers in 41% and antiarrhythmics in 11% of our patients. Nebivolol (DS = 8) frequency was 10% and was the 22nd in our frequency list, while tramadol (DS = 4) occurred in 24% of patients, which is the fifth in the list. A similar study (Jamani et al., 2016) reported the following drugs responsible for the most frequent DDI: oxycodon (15%), metoprolol (14%), clopidogrel (8%) and morphine (6%). These drugs were present in the second half of our frequency list and their frequencies were 6%, 9%, 4% and 2%, respectively. The difference between the two studies may be due to the fact that the above study only included drugs used before AA treatment. In a study about AA and enzalutamide (Vicente-Valor et al., 2021), tamsulosin (17%), tramadol (9%), duloxetine (6%) were the most likely involved medicines in possible DDI with AA. The occurrence of tamsulosin and tramadol in our case has been described above, while the frequency of duloxetine was 1%. The other publications (Graff and Beer, 2014; Benoist et al., 2016; Del Re et al., 2017; Dubinsky et al., 2019) evaluated the potential DDI based on theoretical considerations from the literature. A list of drugs was given with different grades of DDI (Del Re et al., 2017) (drugs not present in our study are in italics and the DS is indicated for other drugs): increased DDI [amiodarone (DS = 23), *flecainide*, *hydrocodone* and propafenone (13)] or moderate (amitriptyline (16), *aripiprazole*, carbamazepine (−5), carvedilol (8), codeine (2.5), *donepezil*, duloxetine (13), *fluoxetine*, *fosamprenavir*, *galantamine*, metoprolol (7), *mexiletine*, *nicardipine*, oxycodone (5), *pioglitazone*, *propranolol*, paroxetine (16), *pimozide*, *risperidone*, *ritonavir*, *trazodone* and venlafaxine (8)]. The grouping is mostly in line with our DS values, but duloxetine and paroxetine could be placed in the “increased” DDI group. The management of anticoagulation in mCRPC patients treated with AA+prednisolone (Dubinsky et al., 2019) was discussed with the emphasis of interactions of AA with warfarin (DS = 13), rivaroxaban (2), endoxaban (0), apixaban (10), dabigatran (0), and low-molecular-weight heparin (1).

In a study of elderly mCRPC patients (Graff and Beer, 2014) the drugs are listed according to the CYP enzyme responsible for DDI with AA: 2D6 substrates [amiodarone (DS = 23), carvedilol (8), *donepezil*, fentanyl (2), *flecainide*, *fluoxetine*, *haloperidol*, *hydrocodone*, metoprolol (7), paroxetine (16), *propranolol*, tamsulosin (4), tramadol (4), *vardenafil* and venlafaxine (8)] and 3A4 inducers [*St John’s Wort* and carbamazepine (−5)]. In another study (Benoist et al., 2016) *flecainide*, *haloperidol*, ketoconazole (4.75), metoprolol (7), oxycodone (5), paroxetine (16), *repaglinide* and rifampicin (−4.25) were mentioned as examples for DDI with AA. In our model, the drugs listed in the two studies above have relatively high or negative DS and may indeed influence the pharmacological characteristics of AA.

Few clinical studies have been conducted on the interaction between individual drugs and AA. One study (Benoist et al., 2018) strongly recommended to replace carbamazepine (DS = −5) during AA treatment. In addition, among the other drugs with negative DS, insulin (−3) and spironolactone (−1) have also been shown to impair the efficacy of AA treatment (Serrano Domingo et al., 2021; Vicente-Valor et al., 2021). The positive effect present in our study of frequently used drugs with positive DS, like vitamin D (5) and pantoprazole (5), which is a proton pump inhibitor (PPI), should be emphasized. The other PPIs, except esomeprazole (4), also possess high DS (9–14) (Table 4). Until now, no interactions are known between AA and PPIs (Uchiyama et al., 2021). Based on our findings, the use of PPIs with high DS may enhances the efficacy of AA. The use of vitamin D (5) plus statins (5–15) was associated with improved OS in mCRPC patients (Carretero-González et al., 2020), but the true role of vitamin D remained unclear, as statins alone improved the survival of patients on AA (Harshman et al., 2017; Gordon et al., 2018; Wilson et al., 2022). The reported role of statins, indomethacin (2.5) (Liu et al., 2017) and leuprorelin (1, but significantly more patients used it in high IDS than in the low IDS group, *p* = 0.028) (Efstathiou et al., 2019) during AA treatment was strengthened by our study. Metformin (1, no difference in frequency in high and low IDS groups) had no effect on efficacy of AA (Mark et al., 2019; Wilson et al., 2022). The results reported for patients using ASI (Wilk et al., 2021) were also found in our study (data not shown), but the longer AA treatment duration was a consequence of significantly more drugs administered (median 10 vs. 7 in our study; *p* = 4.2 × 10^−8^, and (Wilk et al., 2021): 5 vs. 1; *p* <0.01, respectively). On the other hand, ASI includes angiotensin-converting enzyme inhibitors (ACEIs), which have low DS values (0–1, see Table 4), and angiotensin II receptor blockers (ARBs), which have higher DS values (4–13), with the exception of telmisartan (−1). In our study, if ACEIs and ARBs were excluded, the IDS classification (high or low) would change for only 2 and 6 patients, respectively. These data have no statistical significant impact on the final result. Therefore, we conclude that ASIs not *per se*, but high IDS, which is the result of multiple co-medications, influenced TD in our study and probably in the Wilk’s study (Wilk et al., 2021) too. There seems to be a contradiction here, but the deeper analysis presented above showed that the presence of ASI alone does not significantly change the results. In addition, experience in internal medicine or cardiology suggests that ASI users often have ischemic heart disease requiring four to five different types of medication (including statins, diuretics, antiplatelets, proton pump inhibitors, beta-blockers, etc.). This fact may explain the significantly higher number of medications used by ASI users in both studies.

The predictive role of NLR and PLR of mCRPC patients receiving AA was reported in a meta-analysis (Guan et al., 2020) demonstrating significantly (*p* <0.001) longer OS for low NLR and low PLR. In other studies, the progression-free survival was significantly (*p* = 0.008) longer for patients with low NLR (Nieblas-Toscano et al., 2020), but not PLR (Pisano et al., 2021). Our results for AA+prednisolone are consistent with the above findings. However, in the case of AA+dexamethasone the NLR was not predictive, but the high PLR was associated with both the longer TD and OS. In our study, the predictive role of SII, found besides NLR and PLR (Lolli et al., 2016), was not proven. The different results may be caused by the different patient groups (26% of patients had >1 lines chemotherapy and 75% had bone metastases (Lolli et al., 2016) vs. none and 95%, respectively, in our study; etc.) and/or different cut-off level. The effect of PNI on OS (Fan et al., 2017; Küçükarda et al., 2022) was also demonstrated in our study for patients on AA+prednisolone, but no effect of PNI on TD was found. In agreement with the previous findings (Cindolo et al., 2017; Iacovelli et al., 2022), in our study the ≥50% PSA decline was a significant predictor of longer TD and OS.

Since co-medication, which is an independent predictive marker alongside other known predictive markers, it is strongly recommended that co-medication should be considered in all abiraterone studies.

The limitations of our study consist in its retrospective nature, the unknown role of some drugs in steroidogenesis or interaction with CYP450 enzymes, and the arbitrarily-considered scores. Although only a third objective was to investigate the predictive role of hematological markers, a limitation in this case was the lack of data in some patients. It would also be useful to know how different comorbidities affect patients’ survival ab ovo and how AA affects the effectiveness of co-medication. As these are unknown, we suggest that optimization of AA treatment in a personalized way would result in the best clinical outcome.

Despite these limitations, our initial hypothesis proved to be true and our model proved to be useful in demonstrating a strong relationship between IDS and AA efficacy. We found that the higher the IDS (i.e., the more drugs competed with AA for several CYP450 enzyme binding sites), the more AA remained to block androgen synthesis, which ultimately led to an increase in AA efficacy, as reflected in longer TD and OS. This was demonstrated in two different patient groups. Involving physicians from partner specialties, within a given class of drugs, if professionally possible, the drug with the higher DS should be chosen or switched to.

The choice of co-medication should be made in the light of co-morbidities and individual characteristics, and in our study we suggest that for drugs with similar or nearly similar mechanisms of action, the choice of co-medication should be made according to which is more likely to optimize AA treatment. Based on our current data a prospective study aiming to compare the effect of co-medication on survival in AA-treated patients is planned.

In summary we calculated a novel score, called IDS which summarizes all effects of co-medication on AA availability and its biological effects. We hypothesized that if AA binding sites are occupied by the co-drugs, hence more AA would remain to inhibit tumor growth. This assumption was confirmed by our results and in patients who take more drugs representing by high IDS, AA has a better effect and the good pharmacological treatment caused the longer AA treatment. Patients who take few medications deserve special attention. The lack of co-medication may be due to the patient not having co-morbidities, which is less common, or the medication being rejected, not considered important enough or neglected by the patient, so explaining the dual purpose of co-medication is strongly recommended.

## 5 Conclusion

Commonly used drugs in AA-treated mCRPC patients significantly influences the therapeutic effect and OS. The more co-drugs with high DS, the longer the duration of AA treatment and OS. Therefore, a careful medication plan of co-medications is advisable. Prospective studies focusing on commonly used drugs in addition to AA would shed light on the best co-medication strategy. Our model proved to be correct for the drugs used in this cohort confirming the importance of multiple DDI. The model can presumably be generalized to other drugs and other cancer types (or other diseases), but further studies are needed to confirm this.

## Data Availability

The original contributions presented in the study are included in the article/Supplementary Material, further inquiries can be directed to the corresponding author.

## References

[B1] Armstrong C. M. Gao A. C. (2021). Dysregulated androgen synthesis and anti-androgen resistance in advanced prostate cancer. Am. J. Clin. Exp. Urol. 9 (4), 292–300. 34541028 PMC8446765

[B2] Attard G. Merseburger A. S. Arlt W. Sternberg C. N. Feyerabend S. Berruti A. (2019). Assessment of the safety of glucocorticoid regimens in combination with abiraterone acetate for metastatic castration-resistant prostate cancer: A randomized, open-label phase 2 study. JAMA Oncol. 5 (8), 1159–1167. 10.1001/jamaoncol.2019.1011 31246234 PMC6604092

[B3] Benoist G. E. Hendriks R. J. Mulders P. F. Gerritsen W. R. Somford D. M. Schalken J. A. (2016). Pharmacokinetic aspects of the two novel oral drugs used for metastatic castration-resistant prostate cancer: Abiraterone acetate and enzalutamide. Clin. Pharmacokinet. 55 (11), 1369–1380. 10.1007/s40262-016-0403-6 27106175 PMC5069300

[B4] Benoist G. E. van der Doelen M. J. Ter Heine R. van Erp N. P. Mehra N. (2018). A clinically relevant decrease in abiraterone exposure associated with carbamazepine use in a patient with castration-resistant metastatic prostate cancer. Br. J. Clin. Pharmacol. 84 (5), 1064–1067. 10.1111/bcp.13532 29384591 PMC5903233

[B5] Bernard A. Vaccaro N. Acharya M. Jiao J. Monbaliu J. De Vries R. (2015). Impact on abiraterone pharmacokinetics and safety: Open-label drug-drug interaction studies with ketoconazole and rifampicin. Clin. Pharmacol. Drug Dev. 4 (1), 63–73. 10.1002/cpdd.132 27128004

[B6] Biró K. Budai B. Szőnyi M. Küronya Z. Gyergyay F. Nagyiványi K. (2018). Abiraterone acetate + prednisolone treatment beyond prostate specific antigen and radiographic progression in metastatic castration-resistant prostate cancer patients. Urol. Oncol. 36 (2), 81.e1–81. 10.1016/j.urolonc.2017.10.015 29153623

[B7] Bonnet C. Boudou-Rouquette P. Azoulay-Rutman E. Huillard O. Golmard J. L. Carton E. (2017). Potential drug-drug interactions with abiraterone in metastatic castration-resistant prostate cancer patients: A prevalence study in France. Pharmacol 79 (5), 1051–1055. 10.1007/s00280-017-3291-z 28361167

[B8] Carretero-González A. Lora D. Manneh R. Lorente D. Castellano D. de Velasco G. (2020). Combination of statin/vitamin D and metastatic castration-resistant prostate cancer (CRPC): A post hoc analysis of two randomized clinical trials. Clin. Transl. Oncol. 22 (11), 2126–2129. 10.1007/s12094-020-02334-6 32198642

[B9] Cindolo L. Natoli C. De Nunzio C. De Tursi M. Valeriani M. Giacinti S. (2017). Safety and efficacy of abiraterone acetate in chemotherapy-naive patients with metastatic castration-resistant prostate cancer: An Italian multicenter "real life" study. BMC Cancer 17 (1), 753. 10.1186/s12885-017-3755-x 29126389 PMC5681753

[B10] Del Re M. Fogli S. Derosa L. Massari F. De Souza P. Crucitta S. (2017). The role of drug-drug interactions in prostate cancer treatment: Focus on abiraterone acetate/prednisone and enzalutamide. Cancer Treat. Rev. 55, 71–82. 10.1016/j.ctrv.2017.03.001 28340451

[B11] Dubinsky S. Thawer A. McLeod A. G. McFarlane T. R. J. Emmenegger U. (2019). Management of anticoagulation in patients with metastatic castration-resistant prostate cancer receiving abiraterone + prednisone. Support. Care Cancer 27 (9), 3209–3217. 10.1007/s00520-019-04816-y 31073853

[B12] Efsa Scientific Committee Hardy A. Benford D. Halldorsson T. Jeger M. J. Knutsen K. H. (2017). Update: Use of the benchmark dose approach in risk assessment. EFSA J. 15 (1), e04658. 10.2903/j.efsa.2017.4658 32625254 PMC7009819

[B13] Efstathiou E. Davis J. W. Pisters L. Li W. Wen S. McMullin R. P. (2019). Clinical and biological characterisation of localised high-risk prostate cancer: Results of a randomised preoperative study of a luteinising hormone-releasing hormone agonist with or without abiraterone acetate plus prednisone. Eur. Urol. 76 (4), 418–424. 10.1016/j.eururo.2019.05.010 31176622 PMC7205516

[B14] Escudero-Vilaplana V. Collado-Borrell R. Hoyo-Muñoz A. Gimenez-Manzorro A. Calles A. Osorio S. (2020). Potential drug interactions between targeted oral antineoplastic agents and concomitant medication in clinical practice. Expert Opin. Drug. Saf. 19 (8), 1041–1048. 10.1080/14740338.2020.1781089 32529857

[B15] Fan L. Wang X. Chi C. Wang Y. Cai W. Shao X. (2017). Prognostic nutritional index predicts initial response to treatment and prognosis in metastatic castration-resistant prostate cancer patients treated with abiraterone. Prostate 77 (12), 1233–1241. 10.1002/pros.23381 28752926

[B16] Gordon J. A. Buonerba C. Pond G. Crona D. Gillessen S. Lucarelli G. (2018). Statin use and survival in patients with metastatic castration-resistant prostate cancer treated with abiraterone or enzalutamide after docetaxel failure: The international retrospective observational STABEN study. Oncotarget 9 (28), 19861–19873. 10.18632/oncotarget.24888 29731989 PMC5929432

[B17] Graff J. N. Beer T. M. (2014). Pharmacotherapeutic management of metastatic, castration-resistant prostate cancer in the elderly: Focus on non-chemotherapy agents. Drugs Aging 31 (12), 873–882. 10.1007/s40266-014-0224-y 25387443 PMC4418948

[B18] Guan Y. Xiong H. Feng Y. Liao G. Tong T. Pang J. (2020). Revealing the prognostic landscape of neutrophil-to-lymphocyte ratio and platelet-to-lymphocyte ratio in metastatic castration-resistant prostate cancer patients treated with abiraterone or enzalutamide: A meta-analysis. Prostate Cancer Prostatic Dis. 23 (2), 220–231. 10.1038/s41391-020-0209-3 32034294

[B19] Harshman L. C. Werner L. Tripathi A. Wang X. Maughan B. L. Antonarakis E. S. (2017). The impact of statin use on the efficacy of abiraterone acetate in patients with castration-resistant prostate cancer. Prostate 77 (13), 1303–1311. 10.1002/pros.23390 28762529 PMC5811259

[B20] Iacovelli R. Ciccarese C. Caffo O. De Giorgi U. Basso U. Tucci M. (2022). The role of fast and deep PSA response in castration-sensitive prostate cancer. Anticancer Res. 42 (1), 165–172. 10.21873/anticanres.15470 34969722

[B21] Jamani R. Lee E. K. Berry S. R. Saluja R. DeAngelis C. Giotis A. (2016). High prevalence of potential drug-drug interactions in patients with castration-resistant prostate cancer treated with abiraterone acetate. Eur. J. Clin. Pharmacol. 72 (11), 1391–1399. 10.1007/s00228-016-2120-3 27561266

[B22] Küçükarda A. Gökyer A. Gökmen I. Özcan E. Hacıoğlu M. B. Erdoğan B. (2022). Prognostic nutritional index is an independent prognostic factor for treatment response, survival and drug choice in metastatic castration-resistant prostate cancer treated with abiraterone acetate or enzalutamide. Actas Urol. Esp. 46 (5), 301–309. 10.1016/j.acuroe.2021.12.005 35256324

[B23] Liu C. Armstrong C. M. Lou W. Lombard A. Evans C. P. Gao A. C. (2017). Inhibition of AKR1C3 activation overcomes resistance to abiraterone in advanced prostate cancer. Mol. Cancer Ther. 16 (1), 35–44. 10.1158/1535-7163.MCT-16-0186 27794047 PMC5222693

[B24] Lolli C. Caffo O. Scarpi E. Aieta M. Conteduca V. Maines F. (2016). Systemic immune-inflammation index predicts the clinical outcome in patients with mCRPC treated with abiraterone. Front. Pharmacol. 7, 376. 10.3389/fphar.2016.00376 27790145 PMC5062111

[B39] Mark M. Klingbiel D. Mey U. Winterhalder R. Rothermundt C. Gillessen S. (2019). Impact of Addition of Metformin to Abiraterone in Metastatic Castration-Resistant Prostate Cancer Patients With Disease Progressing While Receiving Abiraterone Treatment (MetAb-Pro): phase 2 Pilot Study. Clin. Genitourin. Cancer 17, e323–e328. 10.1016/j.clgc.2018.12.009 30686756

[B25] Nieblas-Toscano D. Arenas-Bonilla A. J. Flores-Martín J. F. Gutiérrez-Tejero F. Velarde-Muñoz C. Ramos-Alaminos C. I. (2020). Role of the neutrophil/lymphocyte ratio in patients with metastatic castration-resistant prostate cancer treated first-line with abiraterone. Actas Urol. Esp. 44 (3), 164–171. 10.1016/j.acuro.2019.11.003 32035807

[B26] Pisano C. Tucci M. Di Stefano R. F. Turco F. Samuelly A. Bungaro M. (2021). Prognostic role of platelet-to-lymphocyte ratio and neutrophil-to-lymphocyte ratio in patients with metastatic castration resistant prostate cancer treated with abiraterone or enzalutamide. Minerva Urol. Nephrol. 73 (6), 803–814. 10.23736/S2724-6051.21.04186-2 33781017

[B27] Serrano Domingo J. J. Alonso Gordoa T. Lorca Álvaro J. Molina-Cerrillo J. Barquín García A. Martínez Sáez O. (2021). The effect of medical and urologic disorders on the survival of patients with metastatic castration resistant prostate cancer treated with abiraterone or enzalutamide. Ther. Adv. Urol. 13, 17562872211043341–13. 10.1177/17562872211043341 34552666 PMC8451255

[B28] Sung H. Ferlay J. Siegel R. L. Laversanne M. Soerjomataram I. Jemal A. (2021). Global cancer statistics 2020: GLOBOCAN estimates of incidence and mortality worldwide for 36 cancers in 185 countries. CA Cancer J. Clin. 71 (3), 209–249. 10.3322/caac.21660 33538338

[B29] Tombal B. (2023). PARP inhibitors: For which mutations and when? https://www.urotoday.com/conference-highlights/eau-annual-congress-2023/eau-2023-prostate-cancer/143097-eau-2023-state-of-the-art-lecture-parp-inhibitors-for-which-mutations-and-when.html (Accessed May 10, 2023).

[B30] Tsoukalas N. Brito-Dellan N. Font C. Butler T. Rojas-Hernandez C. M. Butler T. (2022). Complexity and clinical significance of drug–drug interactions (DDIs) in oncology: Challenging issues in the care of patients regarding cancer-associated thrombosis (CAT). Support. Care Cancer 30 (10), 8559–8573. 10.1007/s00520-022-07235-8 35932318 PMC9512854

[B31] Uchiyama A. A. T. Silva P. A. I. A. Lopes M. S. M. Yen C. T. Ricardo E. D. Mutão T. (2021). Proton pump inhibitors and oncologic treatment efficacy: A practical review of the literature for oncologists. Curr. Oncol. 28 (1), 783–799. 10.3390/curroncol28010076 33546228 PMC7985775

[B32] Venkitaraman R. Thomas K. Huddart R. A. Horwich A. Dearnaley D. P. Parker C. C. (2008). Efficacy of low-dose dexamethasone in castration-refractory prostate cancer. BJU Int. 101 (4), 440–443. 10.1111/j.1464-410X.2007.07261.x 17941935

[B33] Vicente-Valor J. Escudero-Vilaplana V. Collado-Borrell R. Pérez-Ramírez S. Villanueva-Bueno C. Narrillos-Moraza Á. (2021). Potential and real drug interactions in patients treated with abiraterone or enzalutamide in clinical practice. Expert Opin. Drug Metab. Toxicol. 17 (12), 1467–1473. 10.1080/17425255.2021.2027908 35001772

[B34] Wilk M. Waśko-Grabowska A. Skoneczna I. Szmit S. (2021). Angiotensin system inhibitors may improve outcomes of patients with castration-resistant prostate cancer during abiraterone acetate treatment-A cardio-oncology study. Front. Oncol. 11, 664741. 10.3389/fonc.2021.664741 33869068 PMC8047632

[B35] Wilson B. E. Armstrong A. J. de Bono J. Sternberg C. N. Ryan C. J. Scher H. I. (2022). Effects of metformin and statins on outcomes in men with castration-resistant metastatic prostate cancer: Secondary analysis of COU-AA-301 and COU-AA-302. Eur. J. Cancer 170, 296–304. 10.1016/j.ejca.2022.03.042 35568679 PMC9949683

[B36] Wishart D. S. Feunang Y. D. Guo A. C. Lo E. J. Marcu A. Grant J. R. (2018). DrugBank 5.0: A major update to the DrugBank database for 2018. Nucleic Acids Res. 46 (D1), D1074-D1082–D1082. 10.1093/nar/gkx1037 29126136 PMC5753335

[B37] Yang Z. Ni Y. Zhao D. Zhang Y. Wang J. Jiang L. (2021). Corticosteroid switch from prednisone to dexamethasone in metastatic castration-resistant prostate cancer patients with biochemical progression on abiraterone acetate plus prednisone. BMC Cancer 21 (1), 919. 10.1186/s12885-021-08670-2 34388965 PMC8364094

[B38] Zist A. Amir E. Ocana A. F. Seruga B. (2015). Impact of comorbidity on the outcome in men with advanced prostate cancer treated with docetaxel. Radiol. Oncol. 49 (4), 402–408. 10.1515/raon-2015-0038 26834528 PMC4722932

